# Management of minor ailments in a community pharmacy setting: Findings from simulated visits and qualitative study in Gondar town, Ethiopia

**DOI:** 10.1371/journal.pone.0190583

**Published:** 2018-01-04

**Authors:** Asnakew Achaw Ayele, Abebe Basazn Mekuria, Henok Getachew Tegegn, Begashaw Melaku Gebresillassie, Alemayehu Birhane Mekonnen, Daniel Asfaw Erku

**Affiliations:** 1 Department of Clinical Pharmacy, School of Pharmacy, College of Medicine and Health Sciences, University of Gondar, Gondar, Ethiopia; 2 Department of Pharmacology, School of Pharmacy, College of Medicine and Health Sciences, University of Gondar, Gondar, Ethiopia; Universiti Sains Malaysia, MALAYSIA

## Abstract

Community pharmacy professionals are being widely accepted as sources of treatment and advice for managing minor ailments, largely owing to their location at the heart of the community. The aim of the present study was, therefore, to document the involvement of community pharmacy professionals in the management of minor ailments and perceived barriers that limit their provision of such services. Simulated patient (SP) visits combined with a qualitative study using in-depth interviews was conducted among community pharmacy professionals in Gondar town, Northwest Ethiopia. Scenarios of three different minor ailments (uncomplicated upper respiratory tract infection, back pain and acute diarrhea) were selected and results were reported as percentages. Pharmacy professionals were also interviewed about the barriers in the management of minor ailments. Out of 66 simulated visits, 61 cases (92.4%) provided one or more medications to the SPs. Pharmacy professionals in 16 visits asked SPs information on details of symptoms and past medical and medication history. Ibuprofen alone or in combination with paracetamol was the most commonly dispensed analgesics for back pain. Oral rehydration fluid (ORS) with zinc was the most frequently dispensed medication (33.3%) for the management of acute diarrhea followed by mebendazole (23.9%). Moreover, amoxicillin-clavulanic acid capsule (35%) followed by Amoxicillin (25%) were the most commonly dispensed antibiotics for uncomplicated upper respiratory tract infection. Lack of clinical training and poor community awareness towards the role of community pharmacists in the management of minor ailments were the main barriers for the provision of minor ailment management by community pharmacy professionals. Overall, community pharmacists provided inadequate therapy for the simulated minor ailments. Lack of access to clinical training and poor community awareness were the most commonly cited barriers for providing such services. So as to improve community pharmacists’ involvement in managing minor ailments and optimize the contribution of pharmacists, interventions should focus on overcoming the identified barriers.

## Introduction

Community pharmacy professionals, being one of the most reachable healthcare professionals among the community, have great potential as a setting in primary health care services. This characteristic feature of community pharmacies provide a platform for more proactive contribution in self-care and managing a range of minor ailments [1–3].

Minor ailments are generally defined as medical conditions that will resolve on their own and can be reasonably self-diagnosed and self-managed with over-the-counter medications. Examples of minor ailments include headache, back pain, insect bites, heartburn, nasal congestion, etc. [2]. Majority of developed nations, successfully integrated community pharmacy professionals into a variety of public-health programs including providing treatment and advice for managing minor ailments [4]. For instance, the community pharmacist contractual framework of United Kingdom considered ‘minor ailment service as one of the enhanced population health services to be provided by community pharmacy professionals [5, 6]. Similarly, Australian community pharmacy professionals are the most accessible health care professionals for health advice and provision of primary health care services including management of minor ailments [7]. This is in contrast to community pharmacies in developing countries like Ethiopia, where pharmacists’ role is largely confined to the traditional medication dispensing practices and seldom provides such public health services [8, 9]. Furthermore, lack of standard guideline for the management of these conditions is also another problem hindering the realization of such services in developing countries [10]. Different studies were conducted in different parts of Ethiopia regarding self-medication and related issues, but most of these studies utilize consumer’s perspective [11, 12], while the role of community pharmacy professionals in the management of minor ailments and the potential barriers in service delivery are usually overlooked. The aim of the present study was, therefore, to document the involvement of community pharmacists in the management of minor ailments and explore potential barriers and gaps that hindered the provision of such services.

## Materials and methods

### Study design and setting

A simulated patient (SP) visits and qualitative based in-depth interview were conducted in selected community drug retail outlets (CDRSs) located in Gondar town, Ethiopia. Gondar town is located in Amhara regional state, Northwest Ethiopia. According to the 2007 population and housing census report, the town had an estimated population of 206,987 [13]. Gondar town has 20 community pharmacies, 35 drug stores and 2 rural drug vendors. The simulated study was conducted from January 1-February 30, 2016 and the qualitative study was conducted from March 16 to June 1, 2016.

### The simulated patient study

The quantitative simulated patient visit aimed to document the extent of involvement of community pharmacists in the management of minor ailments. In Ethiopia, CDROs are mainly classified into pharmacy (run by a pharmacist with a qualification of bachelor of pharmacy or equivalent), drug shop or drug store (run by pharmacy technicians or druggists with a qualification of diploma in pharmacy or equivalent) and rural drug vendor (run by health assistants or health extension workers). All CDROs in Gondar town were stratified into Arada, Piassa, Azezo and Lideta sub cities (kebeles) and given a random number through MS Excel random number generator (RAND). From a total of 55 CDROs in Gondar, 12 pharmacies and 10 drug stores were selected using a simple random sampling technique and distributed among the above stratified kebeles taking into consideration the number of prescriptions filled per day or patient flow in each kebeles. As each CDRO visited once by each SP, a total of 66 simulated visits were conducted over a 6-weeks period.

### Scenarios and simulated patients

Three different scenarios were developed and enacted by the SPs. The scenarios included common minor ailments, which are considered to be highly frequent (back pain, acute diarrhea and upper respiratory infection). Description of the scenarios employed is summarized in Table 1.

**Table 1:** The scenarios employed in the simulated study, Gondar, 2016.

**Scenario 1: Intermittent back pain**	
The SP is a 32-year-old male with a complaint of intermittent pain in both sides of his lower back. The pharmacist was expected to rule out other medical conditions and advise the SP to take paracetamol, if insufficient advice non-steroidal anti-inflammatory drugs (NSAIDS) or weak opioids, and to visit the nearby hospital if the symptom still persists.	
The pharmacist was given the following information when asked	No previous or current medical condition other than the complaint of intermittent pain.
	The pain started this morning when he bent over to pick up his cloth and it usually worsens during coughing.
	He is not taking any analgesic or other medication for his condition and he is asking for something to alleviate his condition.
**Scenario 2: Acute diarrhea**	
A 29-year-old female simulated patient asked the pharmacist to give her something to alleviate the acute diarrhea suffered by her 4-year-old daughter. The scenario was designed to rule out acute bloody diarrhea (dysentery), persistent diarrhea and diarrhea with malnutrition. The medication recommendation and information provided by the pharmacy personnel, instructions on food and fluid intake and history taking were all recorded.	
The pharmacist was given the following information when asked	No previous or current medical condition other than the complaint of acute watery diarrhea.
	The child is 4 years old
	The diarrhea starts yesterday afternoon (less than 1 day duration)
	There is no blood or mucus in the stool
	No fever
	No one from the family had such complaints.
**Scenario 3: Upper respiratory tract infection (URTI)**	
A 24-year-old male simulated patient asked for a treatment after presenting with a symptom of upper respiratory tract infection (URTI). The SP noted the queries and recommendations provided by the pharmacist including drug allergies, non-pharmacological advices given and medications dispensed.	
The pharmacist was given the following information when asked	No previous or current medical condition other than the complaint of fever, cough and nasal discharge. There is sputum
	The symptoms started 4 days ago

Three graduating (fifth year) undergraduate pharmacy students acted as SPs and each of them were given a specific scenario to play. After a 5-hour detailed discussion and training of each scenario with the SPs, one day was given so that they will be familiar and able to perform the scenarios given. The SPs told not to give and/or ask extra information unless asked in order to make sure that the information provided is consistent across all visits. The data collection tool used by the SPs is presented in supplementary (S1 File).

#### Visit evaluation

All simulated visits were audio recorded so as to avoid counting on the human cognitive processes, which has been cited as a potential shortcoming of the SP methodology [14**]** Instantly after each simulated visit, the SPs filled the collected data in a form containing a check list of items that were intended to assess the overall practice of pharmacists towards management of minor ailments. The check list included questions/patient history asked, medications and/or counselling provided and duration of the visit. Two of the investigators independently compared and validated the data from the check list against audio recordings for the purpose of quality assurance.

### The qualitative study

In the second phase of the study, a qualitative approach was employed with the aim of uncovering the potential barriers to the provision of management services for minor ailments in a community pharmacy setting.

For the purpose of allowing maximum variation [15], sampling and recruitment was done considering types of CDROs, geographical area and pharmacist demographics including age, gender, educational level and experience. Although there is no clear guideline for sample size determination in qualitative study, there is general consensus that the sample size should be determined when theoretical data saturation has been attained [16]. Yet, we predetermined the sample size of community pharmacists for this study so as to allow for the maximal variation mentioned above, and this type of sampling has been recommended in the literature [17]. Accordingly, data were saturated after the 13th interview in our study.

The study participants were recruited through food, medicine and healthcare administration and control authority (FMHACA) of Ethiopia.

Participants were then contacted and invited to take part in the study by telephone. A total of 15 community pharmacy professionals were identified and approached with 13 successfully recruited and interviewed. Semi-structured interviews were conducted by the principal investigator face-to-face at either the respondents’ work place or other agreed location such as coffee houses. The in-depth interview guide was adopted from existing literature with similar topic and includes open-ended questions probing the potential barrier in the management of minor ailments in community pharmacy settings and evolved iteratively as discussions proceeded, along with the use of prompts and cues. The interview took approximately 30 minutes. Minor ailments are operationally defined as conditions that will resolve on their own and can be reasonably self-diagnosed.

### Data analysis and management

Data from the SP visits were entered into and analyzed using Statistical Package for Social Studies (SPSS) version 20 for Windows and results are presented as frequencies and percentages.

For the qualitative study, thematic analysis was carried out, the transcripts and notes were read and annotated repeatedly in order to verify the data for accuracy. The audio-recorded data collected were transcribed and codes were developed by two of the investigators (AAA and DAE) based on the original terms used, and then matched. After all the data have been coded, cutting and pasting of codes was done into piles by code and put them together with other examples of data on the same topic to start looking for patterns across the data. The patterns and relationships found under the themes were the basis for report. Quotes that would help in understanding of the content of the theme or sub-theme were identified. Main themes were illustrated with representative quotations designated as community pharmacist 1 (CP1), CP2……CP3.

## Ethical considerations

Ethical approval to conduct this study was granted by the ethical review committee of School of Pharmacy, University of Gondar with a reference number of UoG-SoP/089/2016. Written informed consent from participants of the qualitative study was also obtained prior to conducting the study. Participant’s information obtained were kept confidential.

## Results

### Outcomes of visit and type of medication (s) recommended

Out of 66 simulated visits (22 each for the three scenarios) presented to CDROs, 61 cases (92.4%) were provided with one or more medications. Before handing out the medications, few community pharmacy professionals (26.2%) asked SPs further details of symptoms and past medical and medication history. Only 9.8% of community pharmacy professionals asked about possible drug allergies and 36.1% of them informed the SPs about the potential side effects of the dispensed medications. The average consultation and/or dispensing times were 2 minutes in drug stores and 3 minutes in pharmacies. The detailed actions and advices provided by the pharmacy professionals is tabulated in Table 2.

**Table 2:** Actions and advice in response to the simulated scenario, Gondar town, Ethiopia, 2016.

Type of action and advice provided	Scenario 1 (Back pain)		Scenario 2 (Diarrhea)		Scenario 3 (URTI)	
	Pharmacies (N = 12)	Drug stores (N = 10)	Pharmacies (N = 12)	Drug stores (N = 10)	Pharmacies (N = 12)	Drug stores (N = 10)
Dispensed medication (s)	11 (91.7%)	9 (90%)	11 (91.7%)	10 (100%)	12 (100%)	8 (80%)
Asks drug allergies	1 (0.91%)	0	2 (18.2%)	0	3 (25%)	0
Instruction on dose and duration	8 (72.7%)	6 (66.7%)	8 (72.7%)	7 (70%)	9 (75%)	5 (62.5%)
Counsel on side effects	5 (45.4%)	2 (22.2%)	4 (36.4%)	3 (30%)	5 (41.7%)	3 (37.5%)
Queries about past medical and medication history	3 (27.3%)	2 (22.2%)	3 (27.3%)	1 (10%)	4 (33.3%)	3 (37.5%)
Advice to visit physician	3 (27.3%)	4 (44.4%)	6 (54.5%)	6 (60%)	4 (33.3%)	3 (37.5%)
Non-pharmacological advice	7 (63.6%)	2 (22.2%)	7 (63.6%)	4 (40%)	5 (41.7%)	3 (37.5%)

**Abbreviations:** CDROs, community drug retail outlets; URTIs, upper respiratory tract infections.

A wide variety of pharmacotherapeutic agents were dispensed and/or recommended for 61 of the simulated scenarios in the pharmacies and drug retail outlets (Table 3). Majority of the medications dispensed for the scenario with back pain were oral analgesics and contained non-steroidal anti-inflammatory drugs (NSAIDS) alone and/or in combination with paracetamol. Ibuprofen alone or in combination with paracetamol was the most commonly dispensed analgesics. A total of five pharmacotherapeutic class of drugs (antiamoebic, antibiotics, anthelmintic, oral rehydration salts (ORS) and zinc) were given for the scenario with acute diarrhea. ORS with zinc was the most frequently dispensed medication (33.3%) followed by mebendazole (23.9%). Finally, Antibiotics were dispensed from 20 out of 22 CDROs in which URTI symptoms were simulated. Amoxicillin-clavulanic acid capsule (35%) followed by Amoxicillin (25%) were the most commonly dispensed antibiotics.

**Table 3:** Medications dispensed in response to the simulated scenario, Gondar town, Ethiopia, 2016.

Medication (s) dispensed	Total	Pharmacies	Drug stores
**Scenario 2 (Back pain), N = 20**			
Paracetamol	3	1	2
Ibuprofen	7	3	4
Diclofenac	3	2	1
Methylsalycylate ointment	1	1	0
NSAIDs plus paracetamol	6	4	2
**Scenario 2 (Diarrhea), N = 21**			
ORS with Zinc	7	4	3
Cotrimoxazole	4	1	3
Metronidazole	4	3	1
Loperamide	1	1	0
Mebendazole	5	2	3
**Scenario 3 (URTI), N = 20**			
Amoxicillin	5	3	2
Amoxicillin-clavulanic acid capsule	7	3	4
Azithromycin	3	3	0
Ciprofloxacin	3	1	2
Amoxicillin plus Azithromycin	2	2	0

**Abbreviation:** ORT: oral rehydration therapy; URTIs, upper respiratory tract infections

### Challenges in the management of minor ailments

The results from the qualitative study depicts an in-depth analysis of barriers that hinders community pharmacists to effectively implement management of minor ailments their practice settings. Eight of the participants were holders of a bachelor degree in pharmacy and the rest of them (5) hold a diploma in pharmacy, with a work experience ranging from 2 year to 15 years (with average of 7 years). Results showed that lack of clinical training and poor community awareness towards the role of community pharmacists in the management of minor ailments were the main barriers and challenges faced by community pharmacists.

### Lack of access to clinical training on the management of minor ailments

Almost all of the participant mentioned lack of training as the most barriers that hinders to provide quality management of minor ailments in community pharmacy settings. According to the participants, much of the clinical trainings are usually provided for health care providers having a direct contact with patients in hospitals such as physicians and nurses, with only few community pharmacists had the chance to take syndrome approach clinical training.

*“…*. *I have taken*, *by chance*, *a symptomatic approach-based clinical training a couple of years ago and it helped me a lot for the management of common ailments in my practice*. *However*, *most of the community pharmacists I have known in Gondar have not taken such training*, *which I believe*, *could be the main reason behind the poor management of minor ailments in many pharmacies and drug stores…*.”

[CP6].

“……. In my point of view, the correct strategy to improve management of common ailments in community pharmacy is that continuous education and trainings. Lack of knowledge is one of the gap I observed. For example, if I have not detail knowledge on how to select appropriate medications for common ailments, I may dispense even wrong medications.”

[CP6].

### Poor community awareness

Poor awareness of the community regarding minor ailment management and the role of community pharmacists in providing these services is another important challenge faced in practice setting. According to the participants, some customers have no awareness regarding generic and brand drugs and they believe that generic drugs are not effective, especially if the product is from local pharmaceutical factory. To meet the needs of the customers, pharmacist provide drugs for common ailments and if the patient’s willingness to pay is less, they will give generic drug with low price as alternative.

…very few customers see community pharmacies and pharmacists as a whole, as a resource to be used for the management of any kind of ailments including minor ailments…besides, most of the patients visiting our pharmacy don’t have any idea about brand/generic medications, and they usually need something better than the usual or generic drugs. For example, patients usually prefer a more potent and brand analgesics than paracetamol…this is also true for antibiotics…instead of amoxicillin, customers prefer to be dispensed amoxicillin-clavulanic acid drug…

## Discussion

The demand for healthcare services within the community settings continues to rise worldwide [18] and government policies promoting community pharmacy-based minor ailment management has been advocated and introduced in many developed countries [19–21]. Studies utilizing a simulated patient visit can be used to assess the actual involvement and quality of care obtained from CDROs within communities. When used in combination with feedback, they can be a very useful means of promoting proper management of minor ailments in community setting [22]. To the best of our knowledge, this is the first attempt to document the involvement of community pharmacy professionals in the management of minor ailments and barriers in the provision of such services in Ethiopia.

In developing country like Ethiopia where the availability of physicians is very limited, enabling community pharmacy professionals to treat minor ailments would have significant advantage from different perspectives such as improving patients’ access to a health care, overall reduction of cost incurred by the patients [4] and alleviate the burden on other health providers including physicians and nurses, allowing them to focus on patients with more complex care needs. Results from our study revealed a high rate of dispensing medications with insignificant queries about past medical and medication history, the potential drug allergies and side effects. Furthermore, most of the medications were dispensed inappropriately. For example, antibiotics dispensed for URTI were inappropriate since the simulated case scenario presented was most likely caused by a virus, not bacterial. Antibiotics are a group of medications which are highly and irrationally dispensed around the globe. Several simulated case studies conducted in many parts of the world have also reported that antibiotics can be obtained with ease from CDROs regardless of laws prohibiting dispensing of these medications without a valid prescription [23–26]. Frequent and irrational use of these medications, which has been known to be the main driver of drug resistance, should be minimized as they may predispose the patient to additional cost due to the need for a broader-spectrum antibiotics and potential hospitalizations for treatment failures [27, 28].

The irrational use of analgesic was also reported in our study including provision of inappropriate dosing and provision of inadequate information. The frequent use of analgesic combinations in our study is one particular issue, raising many safety concerns. Though combining paracetamol with various NSAIDS has been shown to produce better pain relief and control [29], a major fear is unintentional paracetamol overdoses and toxicity by patients, predisposing to potentially deleterious outcomes [30].

Generally, pharmacies performed better than drug stores in most of the simulated case scenarios. This could be due to the fact that drug stores are often staffed by pharmacy technicians, having inadequate medical and pharmacotherapeutic trainings. This may particularly pose a serious problem in the management of minor ailments owing to the easy accessibility and high patronage they receive from the community. Similar studies done elsewhere also reported that drug stores took the lion’s share in the irrational use of medication for several common ailments such as malaria, diarrhea and respiratory infections [31, 32].

Understanding the various barriers for the provision of management service for minor ailments in the community pharmacy settings is a bold step for future improvement in the provision of such services. In our study, lack of access to clinical trainings was the most commonly cited reason as a barrier to the provision of such services. Most of the participants also believed that proper training about how to diagnose, how to select medications for common ailments, how to counsel the customers coming with symptoms have a significant impact on their practice. Treating minor ailments seems like an easy task but well trained pharmacist should be available in community pharmacies to improve the service and to decrease medication therapy related errors. Providing specific trainings that will fill the knowledge and skill gap required for the provision of minor ailment services is recommended both in academia and practice settings as it will result in the delivery of such services in a more skillful way than ever before. Moreover, lack of community awareness on the role of CDROs in the management of minor ailments was also noted as one of the main challenge faced by community pharmacy professionals. It has also been reported that some patients did not accept advice on minor ailments and its management delivered by the pharmacist. A similar study conducted elsewhere also reported that lack of knowledge, lack of confidentiality and privacy and lack of awareness of services are the main barriers preventing young adults accessing pharmacy services [33]. Improving community awareness regarding the role of community pharmacy professionals in management of minor ailments could be one potential solution for proper management of minor ailments. Overcoming the aforesaid barriers also needs restructuring the health care system of the country in an attempt to integrate community pharmacists into the provision of a variety of public health services including minor ailment management. Moreover, considering compensation for service delivery and re-statement of the role and responsibility of community pharmacy professionals may result in a better service delivery.

## Strength and limitations

This study highlights an area of pharmacy practice where there is lack of literature in Ethiopia. All simulated visits were audio recorded so as to avoid counting on the human cognitive processes, which has been cited as a potential shortcoming of the SP methodology. Yet, the study has some limitations that should be taken into account while interpreting the results. It was conducted in community pharmacies serving a relatively homogenous population. Moreover, opinions from the public/patient perspective was not collected which might affect the conclusiveness of some of the findings. Even with the above limitations, this study has significant implications for improving the involvement of community pharmacists in managing minor ailments in community pharmacy settings.

## Conclusions

Community pharmacy professionals provided inadequate therapy for the simulated minor ailments. Lack of access to clinical trainings and poor community awareness were the most commonly cited barriers for providing such services in CDROs. There is a need to ensure that patients know the role of community pharmacy professionals as a care provider for these conditions and the ailments that are appropriate for management in CDROs. Public campaigns and community sensitization are also recommended to improve community’s awareness and boost the uptake of these services. Large scale studies exploring community pharmacy professionals’ involvement in managing minor ailments on a national scale may also be needed to identify more barriers and to better inform regulatory bodies.

## Supporting information

S1 File Data collection tool for the simulated study. (DOCX)

## Abbreviations

CDRSs: community drug retail outlets
CP: community pharmacist
FMHACA: food, medicine and healthcare administration and control authority
NSAIDS: non-steroidal anti-inflammatory drugs
OR: Odds ratio
ORS: oral rehydration salts
SP: simulated patient
SPSS: Statistical Package for the Social Sciences
UoG: University of Gondar
URTI: upper respiratory tract infection
WHO: World health organization
