# Supplementary material for: Management of minor ailments in a community pharmacy setting: Findings from simulated visits and qualitative study in Gondar town, Ethiopia
Source: PLoS One. 2018 Jan 4;13(1):e0190583. doi: 10.1371/journal.pone.0190583 (PMC5754123; doi:10.1371/journal.pone.0190583)
Supplement: S1 File — (DOCX) [file pone.0190583.s001.docx]

**Supplementary data (Data Collection Tool-Simulated Study)**

**Title:** Management of minor ailments in a community pharmacy setting: Findings from simulated visits and qualitative study in Gondar town, Ethiopia

**URTI simulation questionnaire**

| Date: |  |
| --- | --- |
| Pharmacy Name and Full Address: |  |
| Pharmacy No: |  |
| Name of Simulated Patient: |  |

| **Chief compliant:**  You have a cough, running nose and fever of 4 days duration. You go to the pharmacy and ask for treatment for this. You will then wait for the pharmacy personnel to respond now. If he asks you any of the following questions, the following should be your answers. If no questions asked, mark **No** in the last column and skip to the next part. |
| --- |

| **Question** | **Your answer** | **Pharmacist asked question (Y/N)** |
| --- | --- | --- |
| How long since you have the cold/cough/ fever? | 4 days |  |
| How is the intensity of the fever? | I haven’t measured |  |
| Do you have any headache? | Occasionally |  |
| Do you have any sputum? | Yes |  |
| List any other questions he asks you and your response. |  |  |

| If no antibiotics are prescribed, ask the pharmacy personnel to give you a strong medicine for your condition. Record what the pharmacy personnel provided or recommended. If antibiotics are prescribed, write the name and dose of the antibiotic *(fill the following table)* and ask if it is possible to buy today’s dose only for now. |
| --- |

| **Name of drug** | **Particulars he tells you (Y/N** | **Details of particulars (NA if not told)** |
| --- | --- | --- |
| 1 | Dosage |  |
|  | Duration |  |
|  | Side effects, and allergies |  |
| 2 | Dosage |  |
|  | Duration |  |
|  | Side effects, and allergies |  |
| *Record here any other information including non-pharmacologic advice:* | | |

**Acute Child Diarrhea simulation questionnaire**

| Date: |  |
| --- | --- |
| Pharmacy Name and Full Address: |  |
| Pharmacy No: |  |
| Name of Simulated Patient: |  |

| **Chief compliant:**  Your 4 years old child has loose motions since yesterday. You go to the pharmacy and ask for treatment for this. You will then wait for the pharmacy personnel to respond now. If he asks you any of the following questions, the following should be your answers. If no questions asked, mark **No** in the last column and skip to the next part. |
| --- |

| **Question** | **Your answer** | **Pharmacist asked question (Y/N)** |
| --- | --- | --- |
| Frequency of stools | More than 4 times a day |  |
| Intensity of fever | I haven’t measured |  |
| Any abdominal pain | Mild |  |
| How’s the child’s appetite? | Not eating as usual |  |
| Color of stool | Yellow |  |
| Blood/mucus in stool? | Not noticed |  |
| Do you have a prescription? | No |  |
| List any other questions he asks you and your response. |  |  |

| **Treatment and recommendation:**  Record all the medications dispensed and advice given. If no antibiotics are prescribed, ask the pharmacy personnel to give you a strong medicine for your condition. Record what the pharmacy personnel provided or recommended. If antibiotics are prescribed, write the name and dose of the antibiotic *(fill the following table)* and ask if it is possible to buy today’s dose only for now |
| --- |

| **Name of drug** | **Particulars he tells you (Y/N** | **Details of particulars (NA if not told)** |
| --- | --- | --- |
| 1 | Dosage |  |
|  | Duration |  |
|  | Side effects, and allergies |  |
| 2 | Dosage |  |
|  | Duration |  |
|  | Side effects, and allergies |  |
| *Record here any other information including non-pharmacologic advice:* | | |

**Intermittent back pain simulation questionnaire**

| Date: |  |
| --- | --- |
| Pharmacy Name and Full Address: |  |
| Pharmacy No: |  |
| Name of Simulated Patient: |  |

| **Chief compliant:**  You have a complaint of intermittent pain in both sides of your lower back. You go to the pharmacy and ask for treatment for this. You will then wait for the pharmacy personnel to respond now. If he asks you any of the following questions, the following should be your answers. If no questions asked, mark **No** in the last column and skip to the next part. |
| --- |

| **Question** | **Your answer** | **Pharmacist asked question (Y/N)** |
| --- | --- | --- |
| Previous or current medical condition | No |  |
| When the pain did starts? | It starts this morning |  |
| Any aggravating factors? | Gets worse during coughing. |  |
| Did you take any analgesic medication? | No |  |
| Color of stool | Yellow |  |
| Blood/mucus in stool? | Not noticed |  |
| Do you have a prescription? | No |  |
| List any other questions he asks you and your response. |  |  |

| **Treatment and recommendation:**  Record all the medications dispensed and advice given. If the pharmacy personnel dispensed paracetamol, ask the pharmacy personnel to give you a strong medicine for your condition. Record what the pharmacy personnel provided or recommended. |
| --- |

| **Name of drug** | **Particulars he tells you (Y/N** | **Details of particulars (NA if not told)** |
| --- | --- | --- |
| 1 | Dosage |  |
|  | Duration |  |
|  | Side effects, and allergies |  |
| 2 | Dosage |  |
|  | Duration |  |
|  | Side effects, and allergies |  |
| *Record here any other information including non-pharmacologic advice:* | | |
